# Metabolomics meets functional assays: coupling LC–MS and microfluidic cell-based receptor-ligand analyses

**DOI:** 10.1007/s11306-016-1057-y

**Published:** 2016-06-22

**Authors:** M. G. L. Henquet, M. Roelse, R. C. H. de Vos, A. Schipper, G. Polder, N. C. A. de Ruijter, R. D. Hall, M. A. Jongsma

**Affiliations:** BU Bioscience, WageningenUR, Droevendaalsesteeg 1, 6708 PB Wageningen, The Netherlands; Netherlands Metabolomics Centre, Einsteinweg 55, 3335 CC Leiden, The Netherlands; Laboratory of Plant Physiology, WageningenUR, Droevendaalsesteeg 1, 6708 PB Wageningen, The Netherlands; Laboratory of Cell Biology, WageningenUR, Droevendaalsesteeg 1, 6708 PB Wageningen, The Netherlands

**Keywords:** Capsaicin, Receptor-ligand screening, Functional metabolomics, TRPV1, Bioactivity

## Abstract

**Introduction:**

Metabolomics has become a valuable tool in many research areas. However, generating metabolomics-based biochemical profiles without any related bioactivity is only of indirect value in understanding a biological process. Therefore, metabolomics research could greatly benefit from tools that directly determine the bioactivity of the detected compounds.

**Objective:**

We aimed to combine LC–MS metabolomics with a cell based receptor assay. This combination could increase the understanding of biological processes and may provide novel opportunities for functional metabolomics.

**Methods:**

We developed a flow through biosensor with human cells expressing both the TRPV1, a calcium ion channel which responds to capsaicin, and the fluorescent intracellular calcium ion reporter, YC3.6. We have analysed three contrasting *Capsicum* varieties. Two were selected with contrasting degrees of spiciness for characterization by HPLC coupled to high mass resolution MS. Subsequently, the biosensor was then used to link individual pepper compounds with TRPV1 activity.

**Results:**

Among the compounds in the crude pepper fruit extracts, we confirmed capsaicin and also identified both nordihydrocapsaicin and dihydrocapsaicin as true agonists of the TRPV1 receptor. Furthermore, the biosensor was able to detect receptor activity in extracts of both *Capsicum* fruits as well as a commercial product. Sensitivity of the biosensor to this commercial product was similar to the sensory threshold of a human sensory panel.

**Conclusion:**

Our results demonstrate that the TRPV1 biosensor is suitable for detecting bioactive metabolites. Novel opportunities may lie in the development of a continuous functional assay, where the biosensor is directly coupled to the LC–MS.

**Electronic supplementary material:**

The online version of this article (doi:10.1007/s11306-016-1057-y) contains supplementary material, which is available to authorized users.

## Introduction

Metabolomics has become well-established as a valuable approach to investigate the metabolite profiles of living organisms. The technologies used are now firmly embedded in medical, microbial and plant research and are providing detailed snapshots of unique chemical fingerprints of biological responses (Gomez-Casati et al. 2013; Tang 2011). We are however, still facing two significant technical and biological challenges. Firstly, determining the chemical identity (annotation) of the myriad of metabolites now detectable, but often never observed or studied before (Creek et al. 2014). Secondly, determining their potential bioactivity. Generating metabolomics-based biochemical profiles without any associated assessment of the function of the extract or its biochemical component parts is only of indirect value in understanding a biological process (Hall et al. 2011). This paper focuses on the latter aspect by coupling LC–MS mass identification with a flow through bioactivity screening on living cells.

Many metabolites interact with organisms at the cellular level through binding with membrane-bound receptors such as G-protein coupled receptors (GPCRs) and ligand gated ion channels (LGICs) (Regard et al. 2008; Vassilatis et al. 2003). Some of these receptors already have well-described specific ligand interactions. Many others however, are still poorly or even fully uncharacterized. In order to characterize individual receptors and find their equivalent ligands, a multi-well plate, cell-based end point assay is often the approach employed (Tammela et al. 2004; Butler et al. 2014; Johnson et al. 2011; Tu et al. 2010).This screening process could be improved if the LC–MS analysis could be directly coupled to a continuous biological assay. For that, a flow assay format would be required to allow a dynamic monitoring of bioactivity in the course of an analysis and the direct association of a metabolite to a biological response. Here, we have chosen the transient receptor potential channel vanilloid 1 (TRPV1) ion channel as a model sensor. This ion channel has been well-studied for its role in pain and heat perception. TRPV1 is a promiscuous cation channel, with a high preference for calcium, that can be activated by low pH as well as by physical factors such as heat (>42 °C) and membrane depolarization (Chung et al. 2008). However, TRPV1 was first identified as a receptor for capsaicin; a pungent compound found in foods containing chili peppers.

We used capsaicin as standard to test the general properties of the TRPV1 biosensor which uses the intracellular calcium ion sensor, YC3.6, to detect the cytosolic calcium changes that are induced by the activation of the TRPV1 ion channel (Nagai et al. 2004; Roelse et al. 2013). We tested the TRPV1 biosensor using fruit pericarp extracts from three contrasting *Capsicum* varieties. Two were selected with contrasting degrees of spiciness for characterization by HPLC coupled to high mass resolution MS. LC–MS fractions from one pepper extract were screened in a semi-continuous manner, to identify the different bioactive compounds. To confirm biological relevance and complete the proof of concept, we have performed an analysis of the sensation of pungency of tabasco by comparing results from the TRPV1 biosensor with a human taste panel. All findings are discussed in the context of the potential to use this microfluidic biosensor as the first significant step towards an online functional metabolomics tool.

## Materials and methods

### Plasmid construction

The TRPV1 receptor gene was obtained as a cDNA clone from GeneCopoeia (W1312) and cloned into pT2A-YC3.6 without a stop codon using the *AscI* and *Eco*RI sites. The expression vector containing T2A (de Felipe et al. 2006; Hu et al. 2009) and YC3.6 in a single reading frame and the control construct mCherry-T2A-YC3.6 have been previously described (Roelse et al. 2013). Plasmids containing single CFP (cyan fluorescent protein) and YFP (yellow fluorescent protein), pcDNA3-CFP (Addgene plasmid 13030) and pcDNA3-YFP (Addgene plasmid 13033) were used for recording reference spectra.

### Cell culture and transfection

HEK293H cells (ThermoFisher Scientific, 11631-017) were grown as a monolayer in DMEM (Dulbecco’s modified eagle medium, Gibco, 21063-029) containing 1× non-essential amino acids (Gibco, 11140-035), 1× Penicillin–Streptomycin (Gibco, 15140-122) and 10 % FBS (Foetal bovine serum, Gibco, 26140). Cells were transfected with expression vectors pTRPV1-T2A-YC3.6 or pmCherry-T2A-YC3.6 using Effectene transfection reagent (Qiagen, 301425). Selection with Geneticin at 600 µg/ml (Gibco, 10131-027) was initiated 24 h after transfection and maintained for several weeks until stable insertion lines were obtained. From a mixed culture of insertion lines, based on mean fluorescence intensity, a suitable clonal line with intermediate YC3.6 expression was selected for all further experimentation.

### Pepper and tabasco extracts

Three *Capsicum* lines from a large pepper genotype collection (nrs 12, 18 and 28, representing *Capsicum annuum*, *Capsicum chinense* and *Capsicum frutescens*, respectively) were chosen for their reported contrasting levels of capsaicinoids (Wahyuni et al. 2011). Approximately 500 mg frozen pepper pericarp (fruits excluding seeds stored at −80 °C) per variety or 500 mg tabasco suspension (a commercial water and vinegar extract of *C. frutescens* var. tabasco) was diluted to 25 % (w/v) with 1.5 ml 100 % methanol. The samples were sonicated for 15 min, centrifuged, filtered through a 0.2 µM PTFE membrane into an amber glass vial and stored at −20 °C prior to analysis. Aqueous-methanol extracts were analysed by HPLC (see Sect. 2.5) and directly diluted 1:300 or 1:3000 in buffer used in the biosensor assay.

### LC–MS-fractionation of pepper extracts

Our standard LC–MS-based metabolomics profiling platform, composed of a HPLC–PDA–LTQ–Orbitrap FTMS system (Thermo Scientific), was extended with a chip-based nano-electrospray ionization source/fractionation robot (NanoMate Triversa, Advion BioSciences), mounted between the PDA (photodiode array detector) outlet and the inlet of the LTQ-Orbitrap FTMS hybrid system (van der Hooft et al. 2012). The sample injection volume was 10 μl. For chromatographic separation a Luna C18 analytical column (150 × 2.0 mm, 3 μm particle size; Phenomenex) was used with H_2_O and acetonitrile as carriers, both containing 0.1 % v/v formic acid, at a flow rate of 190 µl/min and a column temperature of 40 °C. A linear gradient from 5 to 65 % acetonitrile in MQ water was applied over a period of 45 min, followed by 15 min washing and equilibration before the next injection. Isopropanol (at a flow of 30 µl/min using a syringe pump) was added to the LC eluent flow between the PDA and the NanoMate via a T-junction, to ensure a stable nanospray into the FTMS. The NanoMate source was operated in negative electrospray ionization mode with a HD_A_384 chip at a spray voltage of 1.7 kV. The NanoMate was used in the LC coupling mode with fraction collection. The total flow into the NanoMate (220 µl/min) was split using different capillary tubes for both MS spray (0.480 µl/min) and for 96-wells plate fractionation (219.5 µl/min). LC-fractions were collected every 28 s (i.e., 102.6 μl) into a 96 wells plate (Twin tec, Eppendorf) cooled to 10 °C. After collection, the plate was quickly sealed and stored at −20 °C before further usage. The fractions were assayed on the TRPV1 biosensor as either pools or as separate 96-wells fractions. To firstly create the 8 pools of 12 fractions, half of the well volume (51.3 µl) was used. The pooled and individual fractions were freeze-dried, in order to remove the LC solvent, and then dissolved in 10 µl DMSO (Dimethylsulfoxide). Since 10 µl extract was injected and only half of each fraction was re-dissolved in 10 µl DMSO the sample was two times diluted compared to the original extract. Both pooled (8 pools of 12 fractions) and individual fractions were 200× or 1000× diluted in assay buffer, resulting in a 400× or 2000× dilution from the original extract, depending on their bioactivity.

### Quantification of capsaicinoids in tabasco

Capsaicinoids in the tabasco product and pericarp extracts were quantified essentially as described for pepper pericarp (Wahyuni et al. 2011), by injecting 10 µl of the 75 % methanol extract into a Waters Alliance 2695 HPLC system equipped with a Luna C18 (4.6 × 150 mm; 3 µm particles) at 40 °C and a Waters 2996 PDA detector. A 30 min linear gradient of 50–75 % acetonitrile in MQ water (both acidified with 0.1 % formic acid) was applied to separate the capsaicinoids. The areas under the chromatographic peaks detected at 280 nm were used to calculate the concentrations of the individual and total capsaicinoids, using commercial standards (capsaicin and dihydrocapsaicin) from Sigma (St. Louis, USA).

### TRPV1-biosensor assay

Assays using the TRPV1 cell lines were performed with a flow of 100 µl/min DMEM medium containing 1× non-essential amino acids, 1× Penicillin–Streptomycin and 10 % FBS. Extract and fraction samples, diluted in assay buffer, were injected with an injection loop volume of 50 µl which is equal to the flowcell volume. Data series including the control cell line mCherry-T2A-YC3.6 were generated by mixing the two cell types at a 3:1 ratio of TRPV1:mCherry expressing cells. Both cell lines accurately reported receptor activations that induced subcellular cytoplasmic Ca^2+^ ion rise and fall. Imaging of Cameleon YC3.6 was performed using a Zeiss LSM 510-META 18 confocal laser scanning microscope as previously described in Roelse et al. 2013. Rapid simultaneous acquisition of CFP and YFP emission allowed the use of FRET (Förster resonance energy transfer) ratios as a monitor of ligand induced calcium dynamics. The ImageJ plugin PriFRET was used for the image analysis and pixel-based YFP/CFP ratio calculations according to the following equation:

$$f\left( t \right) = \frac{{{{\mathop \sum \nolimits_{x > q} \left( y \right)} \mathord{\left/ {\vphantom {{\mathop \sum \nolimits_{x > q} \left( y \right)} p}} \right. \kern-0pt} p}}}{{{{\mathop \sum \nolimits_{x > q} \left( c \right)} \mathord{\left/ {\vphantom {{\mathop \sum \nolimits_{x > q} \left( c \right)} p}} \right. \kern-0pt} p}}} - - \mathop \sum \limits_{t = 1}^{n} {{\frac{{{{\mathop \sum \nolimits_{x > q} \left( y \right)} \mathord{\left/ {\vphantom {{\mathop \sum \nolimits_{x > q} \left( y \right)} p}} \right. \kern-0pt} p}}}{{{{\mathop \sum \nolimits_{x > q} \left( c \right)} \mathord{\left/ {\vphantom {{\mathop \sum \nolimits_{x > q} \left( c \right)} p}} \right. \kern-0pt} p}}}} \mathord{\left/ {\vphantom {{\frac{{{{\mathop \sum \nolimits_{x > q} \left( y \right)} \mathord{\left/ {\vphantom {{\mathop \sum \nolimits_{x > q} \left( y \right)} p}} \right. \kern-0pt} p}}}{{{{\mathop \sum \nolimits_{x > q} \left( c \right)} \mathord{\left/ {\vphantom {{\mathop \sum \nolimits_{x > q} \left( c \right)} p}} \right. \kern-0pt} p}}}} {\,n}}} \right. \kern-0pt} {\,n}}$$

where *f* is the YFP/CFP ratio as a function of timestep *t*; *y* and *c* are the pixel values of the YFP and CFP images respectively. One of the images, YFP or CFP, is thresholded, taking only values larger than *q* into account. The threshold value can be set by the user or calculated automatically, where it optimizes for a maximum *f(t)* ratio. The function is normalized by subtracting the average YFP/CFP ratio over the first *n* time steps.

For the experiments described in this paper the threshold *q* was set at zero and the normalization steps *n* at 5. For each experiment, the first injection was done after a minimum of five images. The PriFRET ImageJ plugin is publically available at the WageningenUR website; http://www.wageningenur.nl/en/show/prifret.htm.

### Human sensory panel evaluation

A sensory panel experiment using tabasco was performed by the taste research company “Centrum voor Smaak onderzoek B.V.” in Wageningen, The Netherlands. A panel of 41 humans were given a series of 6 samples, representing a dilution series of five samples from the pure tabasco product and one blank control without tabasco. Dilutions of tabasco were prepared in bottles of 1.5 l commercial mineral water (Spa Blauw). To prevent visual colour and density differences between the tabasco dilutions, 7 g of tomato sauce (Grand Italia Soffritto traditionale) was added to each 1.5 l mineral water. The panel members were given the tabasco dilutions in random order and asked to score the percentage perceived pungency. Individual scoring ranges were first normalized so that the highest and lowest score of each individual would have the same value. All intermediate scores were recalculated proportionally to maintain the relative differences. An ANOVA analysis was performed to determine significance of perceived taste differences between subsequent tabasco dilutions and results were tested for reliability using the Statistical Package for the Social Sciences software (SPSS version 15.0).

## Results and discussion

### Metabolomics and functional assays on pepper extracts

We first analysed pericarp extracts of three different *Capsicum* lines (lines 12, 18 and 28) that were chosen for their contrasting capsaicinoid levels. These three pepper lines were analysed for capsaicinoids by targeted HPLC–PDA (Table 1) and their levels corresponded well to previously determined levels (Wahyuni et al. 2011).

**Table 1:** HPLC quantification of capsaicinoids in crude extracts of three varieties of red chili peppers and a tabasco extract

	Capsaicin (mg/100 g FW)	Dihydrocapsaicin (mg/100 g FW)	Capsaicinoids in extract (µM)^a^
Line 12	n.d.	n.d.	n.d.
Line 18	0.9	n.d.	7.2
Line 28	75.3	25.3	821.8
Tabasco	14.4	9.2	192.6

*FW* fresh weight, *n.d.* not detectable (<0.1 mg/100 g FW)

^a^sum of capsaicin and dihydrocapsaicinin the crude extract

**Fig. 1:**
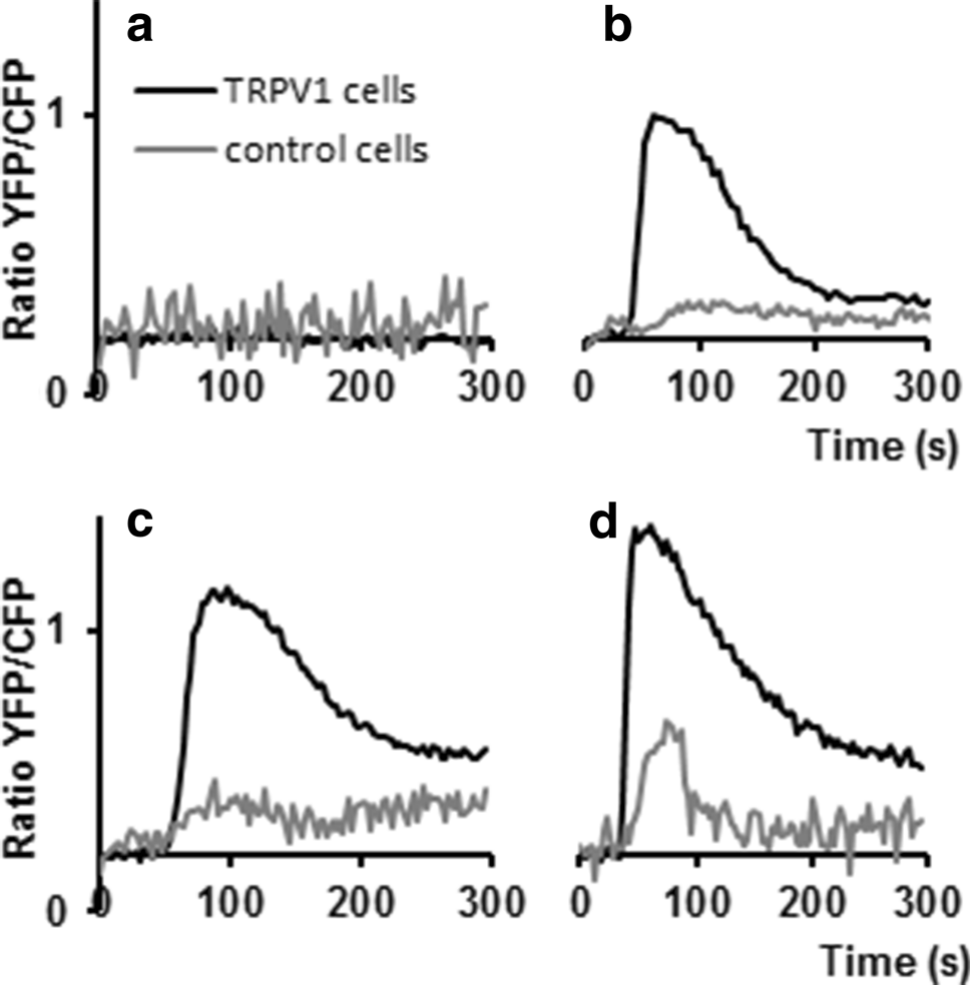
TRPV1 and control cell responses to crude pepper extracts. Cameleon calcium sensor YFP/CFP ratio changes in HEK239 cells were measured after injection of pericarp extracts from three different *pepper lines* with or without capsaicin spikes. The flow-cell contained both TRPV1-expressing cells (*black traces*) and control cells (*grey traces*). **a** Line 12, 300× diluted; **b** line 12, 600× diluted and spiked with 50 nM capsaicin; **c** line 18, 300× diluted; **d** line 28, 3000× diluted

Functional assays of these pepper extracts were subsequently performed with the TRPV1 biosensor. Figure 1 shows the TRPV1 and control cell responses to these pepper extracts. Line 12 showed no response at a 300× dilution of the extract (Fig. 1a), corresponding to the HPLC analysis indicating the undetectable levels of both capsaicin and dihydrocapsaicin (Table 1). When line 12 extract was spiked with 50 nM capsaicin, a normal TRPV1 cell response was observed, showing that the previously observed lack of response is not due to a masking effect by this line 12 (Fig. 1b). In contrast to line 12, line 18 induced a clear response in the TRPV1 cells when exposed to a 300× dilution of the aqueous-methanol extract (Fig. 1c) representing a final concentration of 24 nM total capsaicinoids in the bioassay (Table 1). No response was observed with a 3000× dilution of line 18, representing 2.4 nM of capsaicinoids (data not shown). The most pungent accession, line 28 (Fig. 1d), which contained an approximately 110× higher level of capsaicinoids compared to line 18 (Table 1), gave the highest signal even for the highest dilution tested (3000×). At this dilution, representing 274 nM capsaicinoids, a brief, relative minor response in control cells lacking the exogenous TRPV1 receptor was observed (Fig. 1d). This calcium response in control cells indicates the activation of an endogenous receptor to either the relative high capsaicinoids concentrations or an unknown component only present in the 3000× diluted *Capsicum* line 28 extract. Such responses from control cells were not observed in the 300× diluted extract of line 18, containing 24 nM capsaicinoids. Therefore the control cell response is most likely caused by the high capsaicinoid concentration of line 28.

The specific differential responses of the TRPV1 biosensor to the various *Capsicum* extracts at appropriate dilutions demonstrate that the microfluidic assay can be used on methanol plant extracts dissolved in cell culture medium without a need for further sample clean-up or compound purification. Furthermore, there appears to be a direct and (semi) quantitative relationship between the TRPV1 biosensor response, detected as a change in YFP/CFP fluorescence ratio, and the concentration of ligands present in the extracts (in this case to capsaicinoids), as analytically quantified by HPLC–PDA. This quantitative relationship is shown for pure capsaicin in Sect. 3.3 of the results.

### Metabolomics and functional assays on pepper compounds

**Fig. 2:**
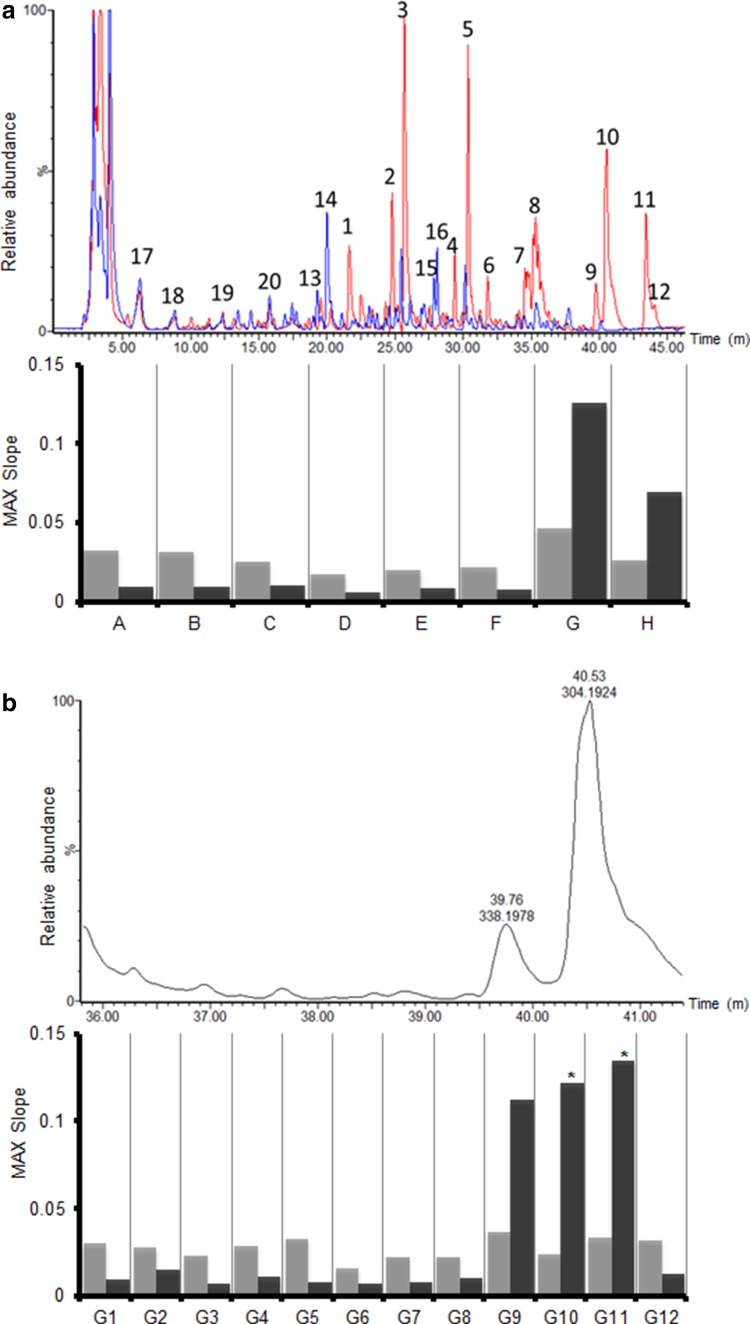

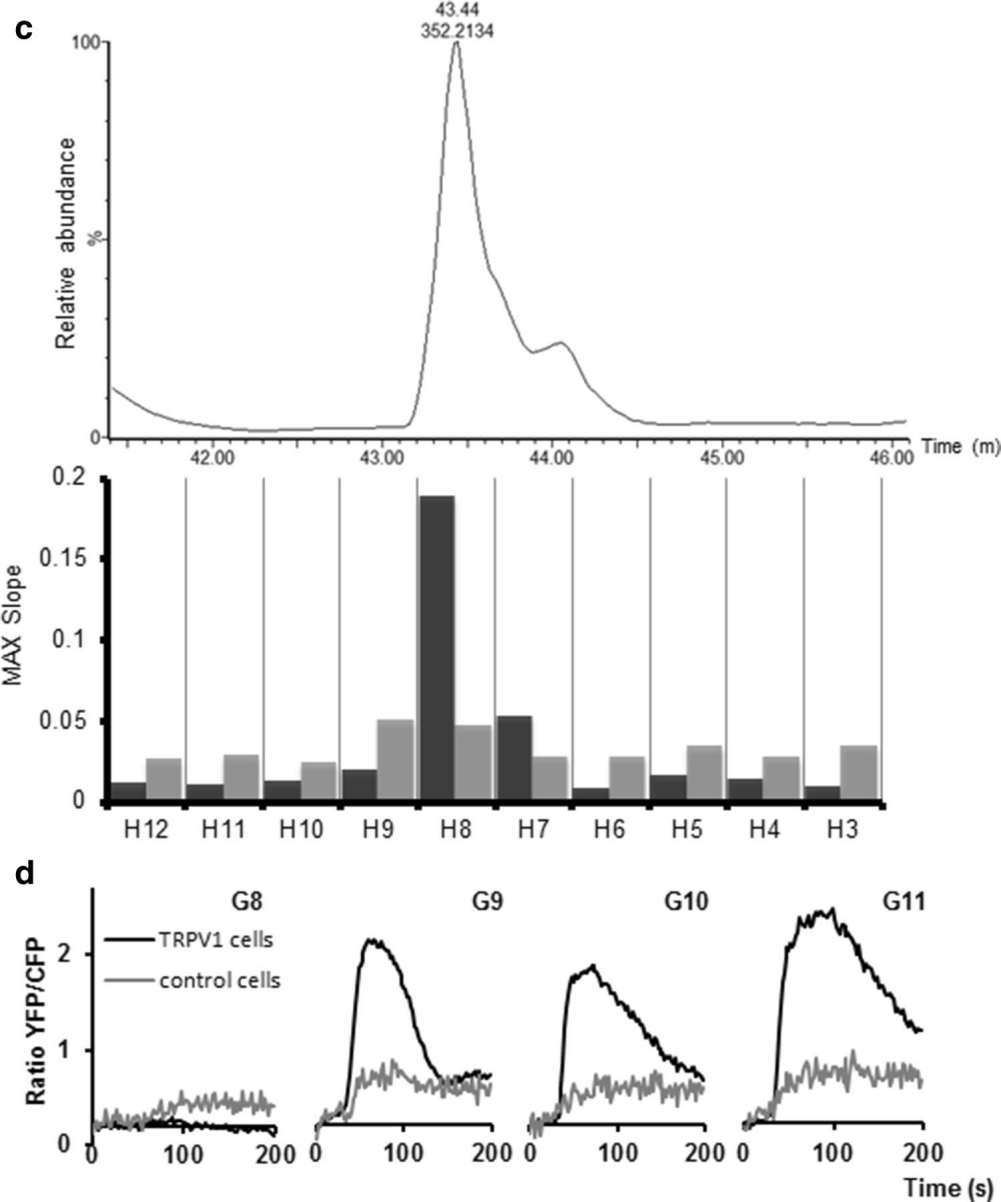
TRPV1 and control cell responses to LC–MS fractions of line 28. **a** Overlaid LC–MS chromatograms (ESI neg mode; base peak intensity) of lines 28 (*red*) and 12 (*blue*). Chromatograms are at the same *Y*-scale to enable direct comparison of the metabolite profiles of both *pepper lines*. The highest peaks were labelled (*1*–*20*) and annotated (Table 2). The NanoMate fractions obtained during LC–MS analysis were subsequently pooled in eight pools of 12 fractions as indicated with *A*–*H*. Responses (maximum slope from moving average of five measurements) to the pooled fractions are displayed below the chromatograms: TRPV1 in *black* and control in *grey bars*. **b** and **c** Details of LC–MS chromatogram and TRPV1 responses of line 28 corresponding to the individual fractions of bioactive pools G (**b**) and H (**c**). All LC fractions were diluted 400×, except fractions *G10* and *G11* (coded with *asterisk*) which were diluted 2000×, as compared to the original pericarp extract. **d** Cameleon calcium sensor YFP/CFP ratio curves; from *left* to *right*: individual fractions *G8*, *G9*, *G10* and *G11* at 400× dilution (*G8* and *G9*) and 2000× dilution (*G10* and *G11*)

The pericarp extracts of the two most contrasting pepper lines 12 and 28 were subjected to HPLC–PDA coupled to both chip-based nano-ESI with accurate mass Orbitrap FTMS and fractionation into 96 wells plates. The overlaid LC–MS chromatograms showed clear differences between these two red pepper lines in their metabolite profiles (Fig. 2a, upper panel). As it was not the aim of the present study to determine all differentially accumulating compounds using comprehensive comparative metabolomics approaches, only the most obvious peaks were specified (Table 2), using the annotations previously reported by (Wahyuni et al. 2013). In general, line 28, which showed highest activity in the TRPV1 bioassay, contained relatively high levels of capsaicinoids, while the TRPV1-inactive line 12, contained relatively high levels of quercetin-type flavonoids.

**Table 2:** Annotation of the most obvious peaks in the pericarp extracts of the two most contrasting pepper lines 12 and 28 using the annotations previously reported by (Wahyuni et al. 2013)

LC–MS				ID level*	mz detected [M-H]-	mz calculated [M-H]-	Mass error (ppm)	Molecular formula	cf. Wahyuni et al. (2013) RT (min) compound ID
Most abundant	peak nr	RT (min)	Compound ID						
Line 28	1	21.6	Unknown	4	841.2058				nd
Line 28	2	24.8	Capsianoside III (FA adduct)	3	1145.5256	1145.5233	2.02	C50H84O26 (+FA)	22.3 Capsianoside III
Line 28	3	25.7	Unknown	4	1185.5195				23.5 unknown
Line 28	4	29.4	Capsianoside VIII (FA adduct)	3	1129.5310	1129.5284	2.33	C50H84O25 (+FA)	27.1 Capsianoside VIII
Line 28	5	30.4	Unknown	4	1169.5247				29.5 unknown
Line 28	6	31.4	Unknown	4	1102.9795^#^				nd
Line 28	7	34.5	Unknown	4	1102.9791^#^				nd
Line 28	8	35.4	Unknown	4	1094.9808^#^				nd
Line 28	9	39.8	Nordihydrocapsaicin (FA adduct)	3	338.1977	338.1973	1.19	C17H27NO3 (+FA)	nd
Line 28	10	40.6	Capsaicin	1	304.1923	304.1918	1.59	C18H27NO3	nd
Line 28	11	43.4	Dihydrocapsaicin (FA adduct)	1	352.2134	352.2129	1.29	C18H29NO3 (+FA)	nd
Line 28	12	43.8	Decanoyl-analogue capsaicin (FA adduct)	3	352.2134	352.2129	1.29	C18H29NO3 (+FA)	nd
			Overlapping with homocapsaicin	3	318.2081	318.2075	1.99	C19H29NO3	nd
Line 12	13	19.3	Quercetin rhamnoside-glucoside	1	609.1467	609.1461	0.97	C27H30O16	18.6 Quercetin rhamnoside-glucoside
Line 12	14	20.0	Quercetin 3-O-rhamnoside	1	447.0937	447.0933	0.93	C21H20O11	19.2 Quercetin 3-O-rhamnoside
Line 12	15	27.9	Unknown	4	1331.5779				28.8 unknown
Line 12	16	28.1	Unknown	4	1185.5203				27.4 unknown
Both	17	6.2	Phenylalanine	1	164.0719	164.0717	1.21	C9H11NO2	nd
Both	18	8.8	Tryptophan	1	203.0827	203.0826	0.49	C11H12N2O2	7.7 tryptophan
Both	19	12.4	Coumaroyl-hexoside	2	325.0933	325.0929	1.26	C15H18O8	11.6coumaric acid-hexose III
Both	20	15.8	Unknown	4	186.1138	186.1136	1.25	C9H17NO3	nd

* ID level (cf. Sumner et al. 2007)

^#^ Double charged molecule

1. Identified compounds

2. Putatively annotated compounds (e.g. without chemical reference standards, based upon physicochemical properties and/or spectral similarity with public/commercial spectral libraries)

3. Putatively characterized compound classes (e.g. based upon characteristic physicochemical properties of a chemical class of compounds, or by spectral similarity to known compounds of a chemical class)

4. Unknown compounds—although unidentified or unclassified these metabolites can still be differentiated and quantified based upon spectral data

To identify which specific compounds in pepper line 28 interact with the TRPV1 ion channel, 96 LC-fractions each of 28 s of elution time were collected in a micro-well plate, using a TriVersa Nanomate fractionation robot, to be tested for their bioactivity. Initially, 8 pools (A–H), each containing 12 consecutive LC-fractions, were semi-continuously tested in the TRPV1 biosensor. The results in Fig. 2a (lower panel) indicate a significant TRPV1-specific response, i.e. a clear signal above the baseline drift of the control cells, for only pools G and H. Subsequently, we analysed the individual fractions from these pools G and H, at 400× or 2000× dilution of the original pericarp extract. The TRPV1 cells responded to the individual fractions G9, G10, G11, H7 and H8 (Fig. 2b, c). The control cells showed a relatively high calcium response with fraction G9, G10 and G11, this control response is caused by the high concentrations of capsaicinoids in these fractions (Fig. 2b, d). In all cases, TRPV1 responding LC-fractions contained compounds detectable by their UV absorbance at 280 nm, indicating the presence of an aromatic (phenolic) backbone. Analysis of the accurate mass data associated with these bioactive LC–MS peaks revealed several known pepper capsaicinoids, including nordihydrocapsaicin, capsaicin, dihydrocapsaicin and homocapsaicin, as well as less, known capsaicin derivatives (Supplementary Table 1). To our knowledge, we have shown that both nordihydrocapsaicin and dihydrocapsaicinare are also true agonists of the human TRPV1 ion channel, in addition to capsaicin.

### Dynamics of the TRPV1 biosensor

**Fig. 3:**
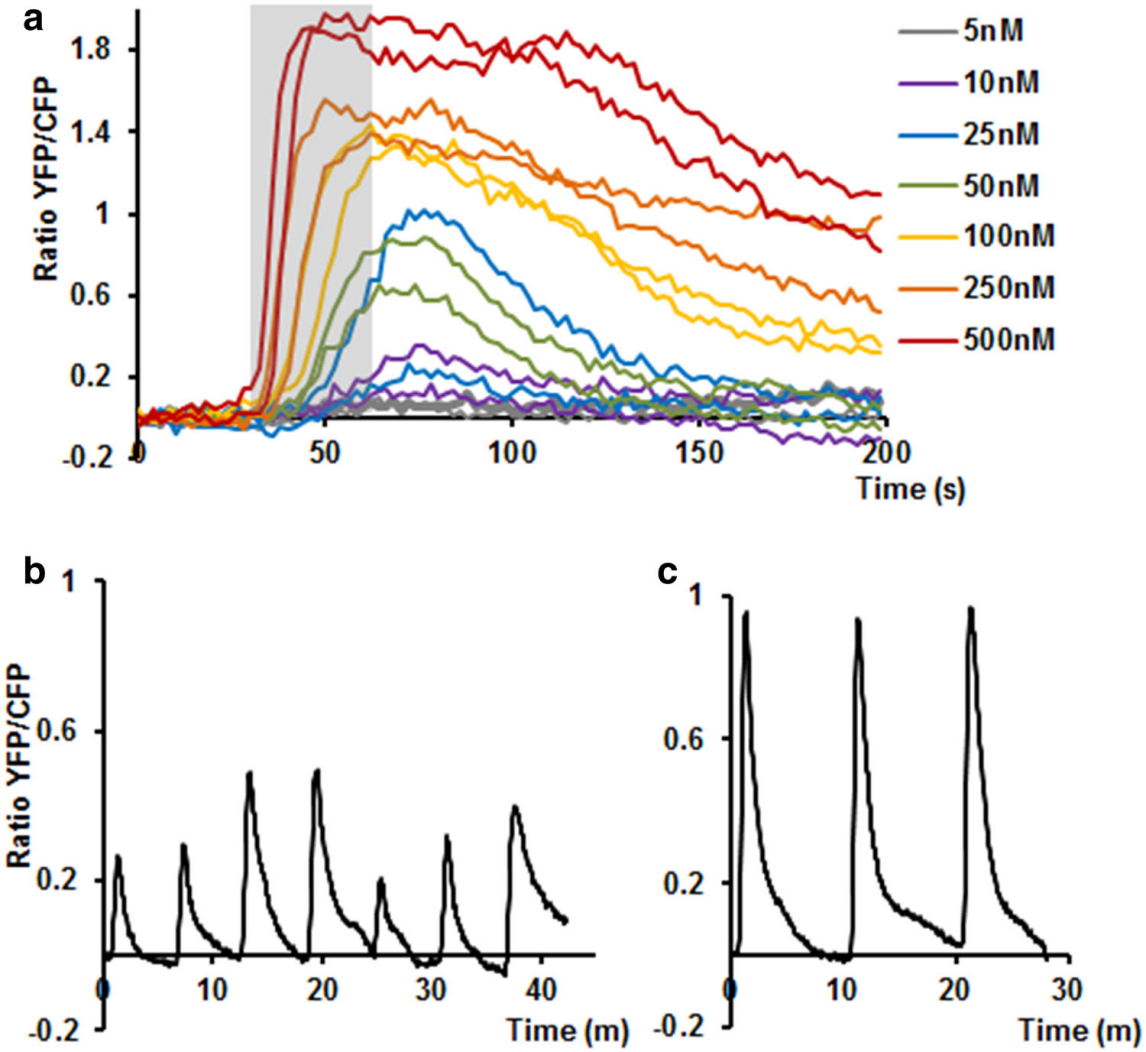
TRPV1 response to capsaicin **a** Cameleon calcium sensor YFP/CFP ratio changes upon injection of increasing capsaicin concentrations. Cell were exposed to 5–500 nM capsaicin for 30 s (indicated by the *grey area*) and their response followed up to 200 s after injection. **b** Cameleon calcium sensor YFP/CFP ratio changes upon repeated exposure to 30 nM capsaicin, by injecting seven times at intervals of 6 min. **c** YFP/CFP ratio changes upon 50 nM doses injected three times at intervals of 10 min

The performance of the biosensor was characterized in more detail by measuring its response to different capsaicin concentrations in the sensor. Each capsaicin concentration was tested twice. Figure 3a shows the TRPV1 responses of the calcium indicator, expressed as the normalized YFP/CFP ratio, to a range of capsaicin concentrations. These data demonstrate that, in our flowcell system, HEK293 cells expressing TRPV1 are able to detect capsaicin in a dose-dependent manner within the concentration range of 5–500 nM. Concentrations above 500 nM were not assayed because these may lead to desensitization of the TRPV1 ion channel (Novakova-Tousova et al. 2007; Touska et al. 2011). To better visualize the dynamics of the system, the exposure time -or dose time- of 30 s is shown as a transparent grey area (Fig. 3a). Related to this dose time we observed four response features: (1) the rate of response, or slope, appeared to be dose dependent. High concentrations of capsaicin showed a fast rise, while low concentrations showed a slower rise. (2) The response duration also appeared to be dose dependent: high concentrations showed a slow return to baseline levels, while low concentrations showed a faster return. (3) At the two highest capsaicin concentrations tested, 250 and 500 nM, the fluorescence ratios remained at their maximum values for about 30 and 50 s, respectively, even after the ligand solution had passed through the flow cell. We call this an elongated response maximum. (4) In contrast, low concentrations of capsaicin, <50 nM, showed a delayed response which only reached its maximum at 50–80 s, i.e. at least 20 s after the ligand solution had already passed through. We call this a delayed response maximum. These observed differences in dynamics give us greater insights into the receptor-ligand interactions at different ligand concentrations and hence represents a significant advantage of such a microfluidic system as compared to the currently used, multi-well, endpoint assay.

Differences in response dynamics of TRPV1 may be explained by existing models for TRPV1 binding stages (Krusek et al. 2004; Jung et al. 1999; Hui et al. 2003; Vyklicky et al. 2003). These models describe partial and full binding stages for capsaicin which affect the opening of the ion channel to partial and full opening, respectively. These binding stages of the TRPV1 ion channel are an effect of the ligand binding to the ligand specific binding sites, of which there are at least two according to Hui et al. 2003. At “high” concentrations of capsaicin, all binding sites on the TRPV1 receptor are occupied resulting in a full opening of the ion channel, while at “low” concentrations there is only a partial occupation of the available binding sites and therefore only a partial ion channel opening. The results we observed in the differential response dynamics of our TRPV1 biosensor at the various ligand concentrations (Fig. 3a) fit well with such model.

Two experiments with repeated capsaicin injections were performed to obtain some insight into TRPV1 response and stability upon repeated exposure to ligands: (1) seven times 30 nM capsaicin at 6 min intervals and (2) three times injections with 50 nM capsaicin at 10 min intervals. At the relative lower concentration and intervals (Fig. 3b), the response tended to vary considerably (standard deviation of 0.103), but without an obvious decline in sensitivity. In contrast, in the second experiment with the longer interval, and higher ligand concentration (Fig. 3c), we observed a highly reproducible pattern with signals fully returning to baseline values and with successive response levels equivalent to the previous ones (standard deviation of 0.014). The relatively high variation at lower concentrations may be due either to the shorter recovery periods (6 min) or may reflect the lower consistency in response at a ligand concentration close to the detection limit (as is also evident in Fig. 3a). Nevertheless, while more detailed and dedicated experiments are required related to the injection frequency, uniformity and stability of the biosensor response, it is clear that this system has potential for sequential use in semi-quantitative/qualitative bioactivity screens.

### Biosensor response benchmarked against a human sensory panel

**Fig. 4:**
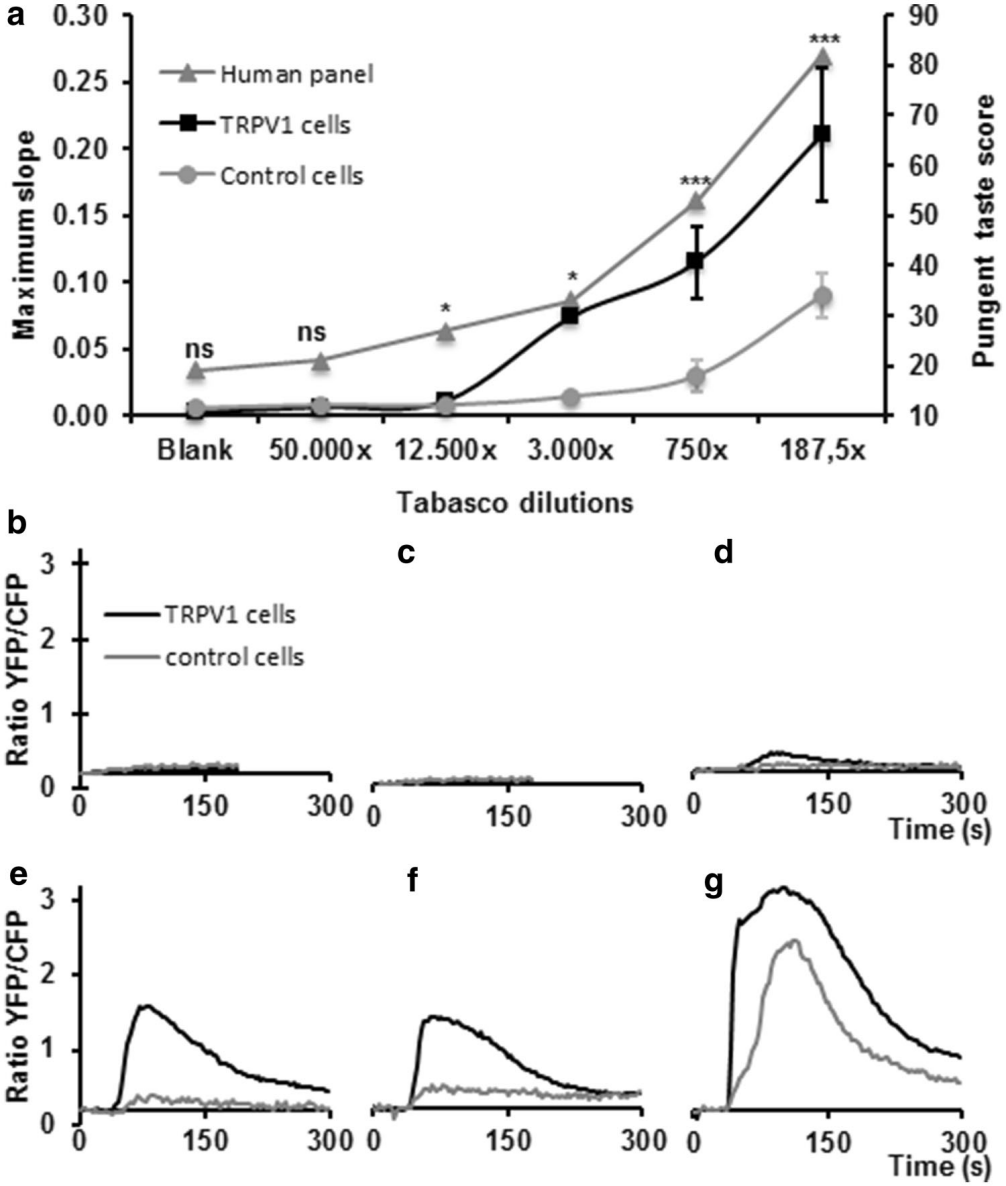
TRPV1 and taste panel responses to tabasco dilutions **a** benchmarking of the TRPV1 biosensor with human test panel results for detecting pungency of tabasco samples. The maximum increase rate of the Cameleon YFP/CFP ratio changes in the biosensor is plotted on the *left axis* (*black squares* TRPV1 cells; *grey circles* control cells). The result of the taste panel assay (*grey triangles*) is plotted on the *right axis* as a pungency score from 0 to 100; *ns* not significant, * = p ≤ 0.05; ** = p ≤ 0.01; *** = p ≤ 0.001. **b**–**g** Cameleon calcium sensor YFP/CFP ratio changes upon increasing tabasco concentrations (water control, 50.000×, 12.000×, 3.000×, 750× and 187.5× for plot **b**–**g**, respectively) in TRPV1 expressing cells (*black curves*) and control cells (*grey curves*)

To allow direct prediction of analysed plant samples to human taste, it is necessary to compare the TRPV1 biosensor system with in vivo taste perception by human individuals. For this reason, the TRPV1 biosensor was benchmarked against a human taste panel using a dilution series (187-50.000×) of a commercial tabasco sauce sample (Fig. 4a, b) which had also been analysed for its capsaicinoid content. The most diluted tabasco sample was indistinguishable from the blank control by both the sensory panel and the biosensor assay. The tabasco pepper has a Scorville scale score ranging from 30.000 to 70.000 SHU, meaning that a dilution in this order of magnitude does not evoke a hot or pungent sensation. The taste panel however, was able to recognize the second-most diluted (12.500×) tabasco sample, just as the biosensor assay was able to detect a small signal for this dilution. This dilution corresponded to a capsaicin concentration of ~15 nM (Table 1). Higher concentrations (lower dilutions) gave increasingly large, responses from the panel as well as in the biosensor assay. With the two highest concentrations in the biosensor assay (equivalent to ~250 and ~1000 nM capsaicin, respectively), the control cells also showed a response, likely caused by either too high concentrations of capsaicinoids or a native response to other compounds present in the tabasco. These results emphasise the importance of using control cells for determining ligand or matrix-induced a specific reactions and the use of the appropriate dilution series for more quantitative analyses of specific bioactivities. Interestingly, the sensitivity thresholds of in vivo (taste panel) and in vitro (receptor microfluidics) measurements were essentially the same, so that these in vitro results can be extrapolated directly to predict human sensory experience.

### Potential for online functional metabolomics

**Fig. 5:**
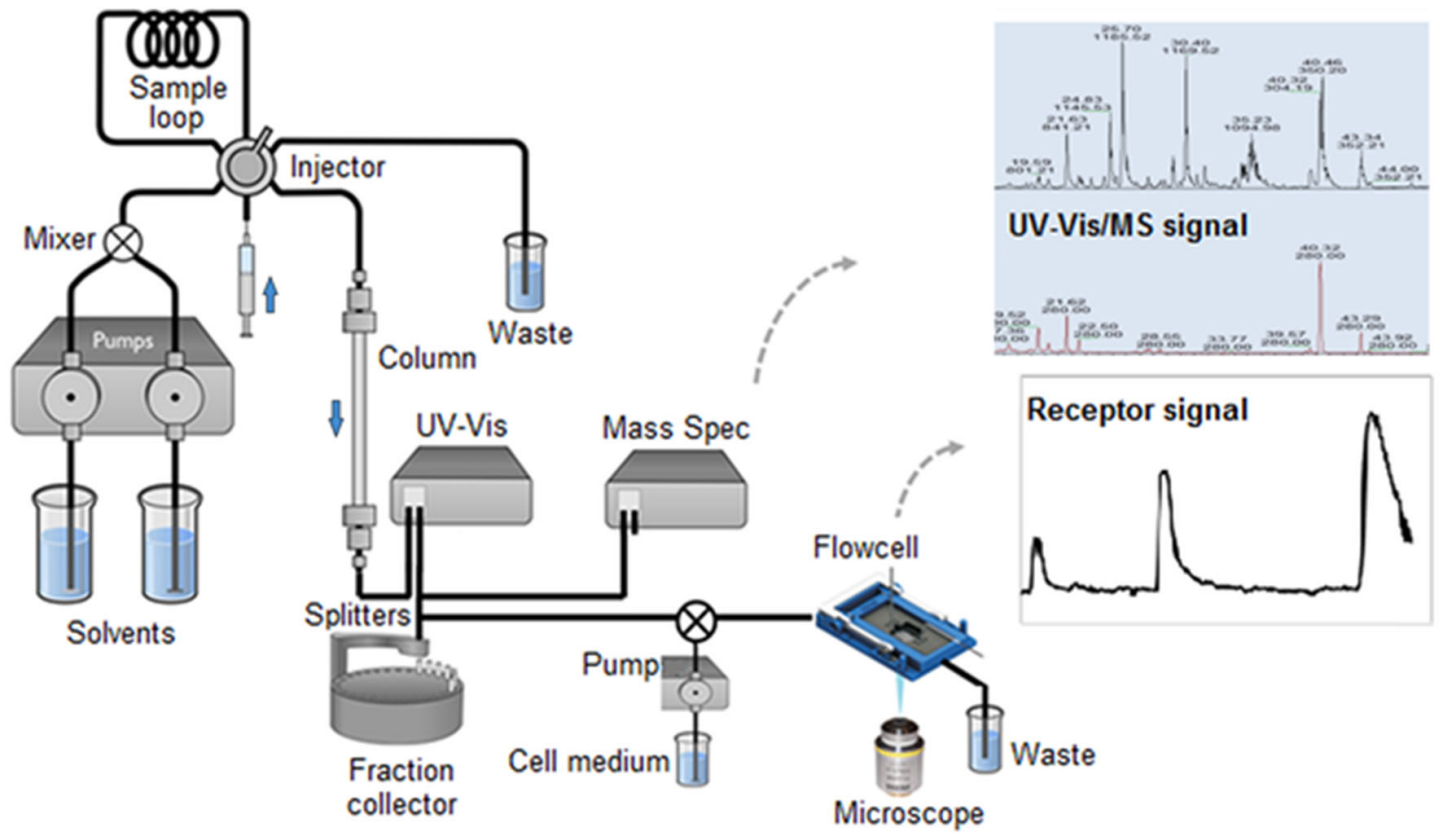
Scheme of an online functional metabolomics setup. The microfluidic flowcell with sensory cells is placed in a microscopic fluorescence detector, which will continuously monitor the cellular response of sample compounds eluting from the LC column. Simultaneous detection of compounds by UV–Vis (*lower chromatogram*) and MS (*upper chromatogram*) can provide identification of bioactive compounds. LC-fractions, preferably separated into individual compounds, are collected as well in order to keep a compound record of the run when further functional analysis or metabolite identification experiments are required. This figure is modified from www.galenica.cl

The results presented were designed to provide a starting point for combining LC–MS analysis with bioactivity assays in series. Existing, online biochemical assays for anti-oxidant detection linked to LC–MS based component identification have already demonstrated the advantages of such online methods (Bountagkidou et al. 2012; Niederlander et al. 2008; Beekwilder et al. 2005). Our study provides the proof of concept that a microfluidic assay with living cells using analytical-scale LC–MS fractions can deliver detectable signals that allow the identification of bioactive molecules present in (crude) plant extracts. The next technical challenge is to design a truly continuous online functional metabolomics application in a flow through system using living cells as the biosensor. A continuous flowcell assay could allow direct measurement of LC output in a single experiment without the need for clean-up/extraction/re-dissolving steps which can lead to compound degradation or modification. Additionally, measuring in a flow-through system readily allows the direct study of receptor dynamics. Figure 5 shows a schematic representation of how such an online LC–MS-based metabolomics-biosensor platform could be realized.

There are, however, some key issues that still need to be addressed to realize such an online biosensor platform. Firstly, there is the issue that the LC solvent maybe incompatible with cell measurements. Acetonitrile or methanol, formic acid, low or high pH, and deviant osmolality are toxic to cells. However, there are several ways to deal with this. Toxicity may be reduced or avoided by significant post column dilution of the sample, prior to cell exposure. Such a dilution is realistic, the plant LC fractions in this study could be diluted with cell culture medium up to 2000 times and still give a clearly measurable sensor response. Alternatively, detoxification of the running buffer may also be achieved by techniques like online post-column solvent evaporation, such as recently developed by (Schoonen et al. 2013). Here the solvent leaving the analytical LC column is evaporated online and the compounds subsequently re-dissolved online in deuterated solvent needed for the NMR analysis.

A second issue is the long-term cell viability and the potential for repeated re-activation of the biosensor cells. In our study with the TRPV1 expressing HEK293 cells, the cells remained vital for at least 1 h at room temperature while continuously imaging the receptor activation. Repeated dosage of up to 50 nM capsaicin produced reproducible activation peaks. At higher dosages of capsaicin, the sensor can easily become saturated. Sample concentration should therefore be carefully monitored and adjusted to avoid receptor over exposure. These are key aspects that need further optimization in the current biosensor setup. However, an investment in time and effort is highly justified as online functional metabolomics using a cell based assay will provide valuable opportunities in automation/high throughput biochemical and bioactivity screening.

## Concluding remarks

Here we have shown that a flowcell containing the HEK239 cell line expressing the TRPV1 ion channel coupled to intracellular calcium sensing, in combination with an appropriate control cell line, provides a sensitive method for semi-continuous functional metabolomics. TRPV1 was activated, resulting in dynamic intracellular calcium levels, both when cells are stimulated with the pure ligand, capsaicin, as well as with crude extracts of capsaicinoid-containing *Capsicum* accessions. Additionally, we have shown that this biosensor compares in sensitivity to a human sensory panel. Furthermore, through application of this system we have been able to determine that, next to the well-known ligand capsaicin, nordihydrocapsaicin and dihydrocapsaicin are true agonists of the human TRPV1 ion channel in hot pepper. A next step will be to tackle the next technical challenges, and meet all biological demands needed to convert this system into a truly continuous and high throughput online functional metabolomics tool.

## Electronic supplementary material

Below is the link to the electronic supplementary material.

Supplementary material 1 (PDF 61 kb)

## References

[CR1] Beekwilder J, Hall RD, de Vos CH (2005). Identification and dietary relevance of antioxidants from raspberry. BioFactors.

[CR2] Bountagkidou O, van der Klift EJC, Tsimidou MZ, Ordoudi SA, van Beek TA (2012). An on-line high performance liquid chromatography-crocin bleaching assay for detection of antioxidants. Journal of Chromatography A.

[CR3] Butler MS, Fontaine F, Cooper MA (2014). Natural product libraries: Assembly, maintenance, and screening. Planta Medica.

[CR4] Chung MK, Guler AD, Caterina MJ (2008). TRPV1 shows dynamic ionic selectivity during agonist stimulation. Nature Neuroscience.

[CR5] Creek DJ, Dunn WB, Fiehn O, Griffin JL, Hall RD, Lei ZT (2014). Metabolite identification: Are you sure? and how do your peers gauge your confidence?. Metabolomics.

[CR6] de Felipe P, Luke GA, Hughes LE, Gani D, Halpin C, Ryan MD (2006). Eunum pluribus: Multiple proteins from a self-processing polyprotein. Trends in biotechnology.

[CR7] Gomez-Casati DF, Zanor MI, Busi MV (2013). Metabolomics in plants and humans: Applications in the prevention and diagnosis of diseases. BioMed research international.

[CR8] Hall RD, Wishart DS, Roessner U (2011). Metabolomics and the move towards biology. Metabolomics.

[CR9] Hu T, Fu Q, Chen P, Zhang K, Guo D (2009). Generation of a stable mammalian cell line for simultaneous expression of multiple genes by using 2A peptide-based lentiviral vector. Biotechnology Letters.

[CR10] Hui K, Liu B, Qin F (2003). Capsaicin activation of the pain receptor, VR1: Multiple open states from both partial and full binding. Biophysical Journal.

[CR11] Johnson TA, Sohn J, Inman WD, Estee SA, Loveridge ST, Vervoort HC (2011). Natural product libraries to accelerate the high-throughput discovery of therapeutic leads. Journal of Natural Products.

[CR12] Jung J, Hwang SW, Kwak J, Lee SY, Kang CJ, Kim WB (1999). Capsaicin binds to the intracellular domain of the capsaicin-activated ion channel. Journal of Neuroscience.

[CR13] Krusek J, Dittert I, Hendrych T, Hnik P, Horak M, Petrovic M (2004). Activation and modulation of ligand-gated ion channels. Physiological Research.

[CR14] Nagai T, Yamada S, Tominaga T, Ichikawa M, Miyawaki A (2004). Expanded dynamic range of fluorescent indicators for Ca(2+) by circularly permuted yellow fluorescent proteins. Proceedings of the National Academy of Sciences of the United States of America.

[CR15] Niederlander HA, van Beek TA, Bartasiute A, Koleva II (2008). Antioxidant activity assays on-line with liquid chromatography. Journal of Chromatography A.

[CR16] Novakova-Tousova K, Vyklicky L, Susankova K, Benedikt J, Samad A, Teisinger J (2007). Functional changes in the vanilloid receptor subtype 1 channel during and after acute desensitization. Neuroscience.

[CR17] Regard JB, Sato IT, Coughlin SR (2008). Anatomical profiling of G protein-coupled receptor expression. Cell.

[CR18] Roelse M, de Ruijter NC, Vrouwe EX, Jongsma MA (2013). A generic microfluidic biosensor of G protein-coupled receptor activation-monitoring cytoplasmic [Ca(2+)] changes in human HEK293 cells. Biosensors & Bioelectronics.

[CR19] Schoonen JW, Vulto P, de Roo N, van Duynhoven J, van der Linden H, Hankemeier T (2013). Solvent exchange module for LC-NMR hyphenation using machine vision-controlled droplet evaporation. Analytical Chemistry.

[CR20] Sumner LW, Amberg A, Barrett D, Beale MH, Beger R, Daykin CA (2007). Proposed minimum reporting standards for chemical analysis chemical analysis working group (CAWG) metabolomics standards initiative (MSI). Metabolomics.

[CR21] Tammela P, Wennberg T, Vuorela H, Vuorela P (2004). HPLC micro-fractionation coupled to a cell-based assay for automated on-line primary screening of calcium antagonistic components in plant extracts. Analytical and Bioanalytical Chemistry.

[CR22] Tang J (2011). Microbial metabolomics. Current Genomics.

[CR23] Touska F, Marsakova L, Teisinger J, Vlachova V (2011). A “cute” desensitization of TRPV1. Current Pharmaceutical Biotechnology.

[CR24] Tu Y, Jeffries C, Ruan H, Nelson C, Smithson D, Shelat AA (2010). Automated high-throughput system to fractionate plant natural products for drug discovery. Journal of Natural Products.

[CR25] van der Hooft JJ, de Vos RC, Mihaleva V, Bino RJ, Ridder L, de Roo N (2012). Structural elucidation and quantification of phenolic conjugates present in human urine after tea intake. Analytical Chemistry.

[CR26] Vassilatis DK, Hohmann JG, Zeng H, Li F, Ranchalis JE, Mortrud MT (2003). The G protein-coupled receptor repertoires of human and mouse. Proceedings of the National Academy of Sciences of the United States of America.

[CR27] Vyklicky L, Lyfenko A, Kuffler DP, Vlachova V (2003). Vanilloid receptor TRPV1 is not activated by vanilloids applied intracellularly. NeuroReport.

[CR28] Wahyuni Y, Ballester AR, Sudarmonowati E, Bino RJ, Bovy AG (2011). Metabolite biodiversity in pepper (*capsicum*) fruits of thirty-two diverse accessions: Variation in health-related compounds and implications for breeding. Phytochemistry.

[CR29] Wahyuni Y, Ballester AR, Tikunov Y, de Vos RC, Pelgrom KT, Maharijaya A (2013). Metabolomics and molecular marker analysis to explore pepper (*Capsicum* sp.) biodiversity. Metabolomics.

