# Supplementary material for: Metabolomics meets functional assays: coupling LC–MS and microfluidic cell-based receptor-ligand analyses
Source: Metabolomics. 2016 Jun 22;12:115. doi: 10.1007/s11306-016-1057-y (PMC4917570; doi:10.1007/s11306-016-1057-y)
Supplement: Supplementary file 1 — Supplementary material 1 (PDF 61 kb) [file 11306_2016_1057_MOESM1_ESM.pdf]

compound name	capsaicin analogue	ID level*	LC-UV RT (min) line 12	LC-UV RT (min) line 28	area 280nm line 28	LC-MS RT (min) line 12	LC-MS RT (min) line 28	mz detected [M-H]-	mz calculated [M-H]-	molecular formula	mass error (ppm)
Nornorcapsaicin	6-Methyl-4*E* -heptenoyl	3	nd	32.62	541,230	nd	33.29	276.16101	276.16052	C16H23NO3	1.79
	Octanoyl	3	nd	35.75	592,490	nd	36.44	278.17665	278.17617	C16H25NO3	1.73
Norcapsaicin	7-Methyl-5*E* -octenoyl	3	nd	36.34	147,880	nd	37.01	290.17647	290.17617	C17H25NO3	1.05
	6-Nonenoyl	3	nd	36.57	54,880	nd	37.26	290.17638	290.17617	C17H25NO3	0.72
Nordihydrocapsaicin	7-Methyloctanoyl	3	nd	39.06	3,318,300	39.67	39.76	292.19233	292.19182	C17H27NO3	1.76
ω-Hydroxycapsaicin	9''-Hydroxy	3	nd	39.06	3,318,300	nd	39.94	320.18721	320.18673	C18H27NO4	1.51
Nonivamide	Nonanoyl	3	nd	39.70	overlap with capsaicin	nd	40.33	292.19238	292.19182	C17H27NO3	1.92
Capsaicin^		1	nd	39.82	58,457,950	40.38	40.47	304.19228	304.19182	C18H27NO3	1.52
Zucapsaicin	(*Z*)-isomer	3	nd	40.20	988,180	nd	40.82	304.19229	304.19182	C18H27NO3	1.55
Dihydrocapsaicin	8-Methyl-nonanoyl	1	nd	42.73	14,078,620	43.25	43.38	306.20792	306.20747	C18H29NO3	1.46
Homocapsaicin	9-Methyl-7*E* -decenoyl	3	nd	43.34	2,576,880	nd	43.95	318.20809	318.20747	C19H29NO3	1.97
	Decanoyl	3	nd	43.34	2,576,880	nd	43.98	306.20807	306.20747	C18H29NO3	1.83
	10-Methyl-8*E* -undecenoyl	3	nd	nd		nd	46.13	332.22333	332.22312	C20H31NO3	0.52
Homodihydrocapsaicin	9-Methyldecanoyl	3	nd	46.27	681,430	nd	46.98	320.22344	320.22312	C19H31NO3	1.01
			nd	46.27	681,430	nd	47.14	332.22346	332.22312	C20H31NO3	1.04
Capsaicin glucoside			nd	nd		nd	nd	nd	466.24464	C24H37NO8	
Dihydrocapsaicin glucoside			nd	nd		nd	nd	nd	468.26029	C24H39NO8	

Capsaicin^: N-[(4-Hydroxy-3-methoxyphenyl)methyl]-8-methyl-6-nonenamide

ID level* (cf. Sumner et al. 2007)

1. Identified compounds
2. Putatively annotated compounds (e.g. without chemical reference standards, based upon physicochemical properties and/or spectral similarity with public/commercial spectral libraries)
3. Putatively characterized compound classes (e.g. based upon characteristic physicochemical properties of a chemical class of compounds, or by spectral similarity to known compounds of a chemical class)
4. Unknown compounds—although unidentified or unclassified these metabolites can still be differentiated and quantified based upon spectral data
